# Cost-effectiveness of two online interventions supporting self-care for eczema for parents/carers and young people

**DOI:** 10.1007/s10198-023-01649-9

**Published:** 2024-01-09

**Authors:** Tracey H. Sach, Mary Onoja, Holly Clarke, Miriam Santer, Ingrid Muller, Taeko Becque, Beth Stuart, Julie Hooper, Mary Steele, Sylvia Wilczynska, Matthew J. Ridd, Amanda Roberts, Amina Ahmed, Lucy Yardley, Paul Little, Kate Greenwell, Katy Sivyer, Jacqui Nuttall, Gareth Griffiths, Sandra Lawton, Sinéad M. Langan, Laura Howells, Paul Leighton, Hywel C. Williams, Kim S. Thomas

**Affiliations:** 1 Health Economics Group, Norwich Medical School, University of East Anglia, Norwich Research Park, Norwich, NR4 7TJ UK; 2 Primary Care Research Centre, Faculty of Medicine, Population Sciences and Medical Education, University of Southampton, Aldermoor Close, Southampton, SO16 5ST UK; 3 Pragmatic Trials Unit, Wolfson Institute of Population Health, Queen Mary University of London, Yvonne Carter Building, 58 Turner Street, London, E1 2AB UK; 4 King’s Clinical Trial Unit, King’s College London, Institute of Psychiatry, Psychology and Neuroscience, 16 De Crespigny Park, London, SE5 8AB UK; 5 Centre for Academic Primary Care, Population Health Sciences, Bristol Medical School, University of Bristol, Canynge Hall, 39 Whatley Road, Bristol, BS8 2PS UK; 6 Patient and Public Contributor, Centre of Evidence Based Dermatology, School of Medicine, University of Nottingham, Applied Health Services Research Building (Building Number 42), University Park, Nottingham, NG7 2RD UK; 7 Centre for Clinical and Community Applications of Health Psychology, Faculty of Environmental and Life Sciences, University of Southampton, Southampton, UK; 8 Southampton Clinical Trial Unit, University of Southampton and University Hospital Southampton NHS Foundation Trust, Southampton, UK; 9 Dermatology, The Rotherham NHS Foundation Trust, Rotherham, UK; 10 Department of Non-Communicable Disease Epidemiology, London School of Hygiene and Tropical Medicine, London, UK; 11 Centre of Evidence Based Dermatology, School of Medicine, University of Nottingham, Applied Health Services Research Building (Building Number 42), University Park, Nottingham, NG7 2RD UK; 12 School of Psychological Science, University of Bristol, 12A Priory Rd, Bristol, BS8 1TU UK

**Keywords:** Economic evaluation, Cost-effectiveness, Atopic eczema, Atopic dermatitis, Online interventions, D610, I100

## Abstract

**Objective:**

To estimate the cost-effectiveness of online behavioral interventions (EczemaCareOnline.org.uk) designed to support eczema self-care management for parents/carers and young people from an NHS perspective.

**Methods:**

Two within-trial economic evaluations, using regression-based approaches, adjusting for baseline and pre-specified confounder variables, were undertaken alongside two independent, pragmatic, parallel group, unmasked randomized controlled trials, recruiting through primary care. Trial 1 recruited 340 parents/carers of children aged 0–12 years and Trial 2 337 young people aged 13–25 years with eczema scored ≥ 5 on Patient-Oriented Eczema Measure (POEM). Participants were randomized (1:1) to online intervention plus usual care or usual care alone. Resource use, collected via medical notes review, was valued using published unit costs in UK £Sterling 2021. Quality-of-life was elicited using proxy CHU-9D in Trial 1 and self-report EQ-5D-5L in Trial 2.

**Results:**

The intervention was dominant (cost saving and more effective) with a high probability of cost-effectiveness (> 68%) in most analyses. The exception was the complete case cost–utility analysis for Trial 1 (omitting participants with children aged < 2), with adjusted incremental cost savings of -£34.15 (95% CI  – 104.54 to 36.24) and incremental QALYs of – 0.003 (95% CI  – 0.021 to 0.015) producing an incremental cost per QALY of £12,466. In the secondary combined (Trials 1 and 2) cost-effectiveness analysis, the adjusted incremental cost was -£20.35 (95% CI  – 55.41 to 14.70) with incremental success (≥ 2-point change on POEM) of 10.3% (95% CI 2.3–18.1%).

**Conclusion:**

The free at point of use online eczema self-management intervention was low cost to run and cost-effective.

**Trial registration:**

This trial was registered prospectively with the ISRCTN registry (ISRCTN79282252). URL www.EczemaCareOnline.org.uk.

**Supplementary Information:**

The online version contains supplementary material available at 10.1007/s10198-023-01649-9.

## Introduction

Atopic eczema (atopic dermatitis), referred to here as eczema, is the most common form of the chronic, inflammatory, and relapsing skin disease whose symptoms (inflamed skin, intense itch, disruption of the skin barrier) result in discomfort, infection, and bleeding of the skin [1]. Eczema substantially impacts an individual’s quality of life through sleep deprivation, psychological effects, regular healthcare visits, and employment loss [2]. These impacts have the potential of increasing the cost implications for patients, their families, and the health system [3].

Research on eczema education and self-management interventions is limited [4]. Jackson et al. [5] report on a structured, theory-based, nurse-led group intervention for parents of children with eczema that was delivered via a 2-h session every week for 3 weeks. The authors provide a crude estimate of the intervention delivery cost as £120 per family. The only economic evaluation reported [6] for a 12-week group educational program reports little detail but concluded that the intervention was not cost-effective at 6 months. Further research on the cost-effectiveness of different delivery models has been recommended [4].

Given this recommendation and the absence of published evidence on the cost-effectiveness of online self-management eczema interventions, this study aimed to conduct two within-trial economic evaluations to determine the cost-effectiveness of two online interventions supporting self-care for eczema (Eczema Care Online (ECO) for parent/carers and ECO for young people) compared to usual care alone from an NHS perspective [7, 8].

## Materials and methods

The within-trial economic analyses used individual participant level data from the ECO trials in which participants were followed up for 12 months [7]. The evaluation was undertaken in accordance with published guidelines for the economic evaluation of health care interventions [9–13]. Since the trials were conducted in the UK which has a national health service (NHS), providing publicly funded healthcare which is largely free of charge at the point of use, the analysis took an NHS perspective. This is in keeping with the NICE reference case [13] since the clinical team felt that Personal Social Services (PSS) were unlikely to be relevant to those with eczema.

The ECO trials have been described in detail elsewhere [7, 8], but in brief, participants were recruited from 98 general practices in England, using GP records to identify people with eczema who had obtained a prescription for eczema treatment in the past 12 months. Participants had a POEM score ≥ 5 (indicating mild to severe eczema but excluding those with very mild or inactive eczema to avoid floor effects). In Trial 1, participants were included if they were either a parent or carer of a child aged 0–12 years and in Trial 2, if they were aged 13–25 years. Participants were randomly assigned (1:1) to one of two groups (online intervention with usual care or usual care alone).

### Interventions

#### Usual care group

Participants randomized to the usual care group continued to receive their usual medical advice and prescriptions for eczema. They were able to seek online support for their eczema and were recommended to use a standard informational website (National Eczema Society. https://eczema.org/). They did not have access to the intervention (website) during their participation in the trial but were given access on completion of the 12-month follow-up period.

#### Intervention plus usual care group

Intervention participants received usual care as described above plus signposting to the online behavioral intervention. Minimum engagement with the intervention was defined as the completion of core material on getting control (flare control creams) and keeping control (use of emollients). Interventions are described fully in the development papers [14, 15].

### Resource use and costs

In line with the chosen perspective, the base case captures the ongoing intervention costs to the NHS and the participant’s wider use of the NHS (primary and secondary health care visits and prescriptions) as related to eczema.

Only intervention resources incurred in running the website were included in the analysis. This included server costs to host intervention, domain name and emails. The maintenance costs were apportioned to participants equally although if rolled out, the per-participant maintenance cost is likely to be very small given the potential number of intervention users. Costs associated exclusively with research activities, such as the cost of developing the intervention (including professional and patient time, time to create content e.g., audio–visual features, and the programming costs), were not included in the economic analysis in line with other economic evaluations of digital interventions, as they were not funded by the NHS but are reported separately as recommended [16].

All resources used (medication use and service use) were collected via medical notes review (see Appendix S2) at GP practices for the entire 12-month study period plus a 3-month pre-baseline period to enable adjustments for baseline costs in adjusted analyses. Resources were valued using published UK unit costs (in £Sterling 2021)[17–19].

### Outcomes

#### Health-related quality of life

Quality-adjusted life years (QALYs) were estimated via utility scores obtained using the proxy CHU-9D in Trial 1 and the self-complete EQ-5D-5L for Trial 2. Utility measurements were collected at baseline, 24 and 52 weeks via online questionnaire. The CHU-9D is a pediatric generic preference-based measure of health-related quality of life suitable for 7- to 17-year-olds, with a proxy version available for 5–6-year-olds. Additional guidance, provided by the developer, aimed at helping parents of preschool-aged children complete the instrument was used for children aged 2 to 4 years in Trial 1. The CHU-9D consists of 9 questions (worry, sadness, pain, tiredness, annoyance, school, sleep, daily routine, and activities) with 5-response levels per question (doesn’t feel/ a little bit/ a bit/ quite/ very or as no problems/ a few problems /some problems/ many problems/ can’t do) [20]. Responses to the CHU-9D were converted to utility scores using the UK valuation set [20]. The CHU-9D was valued by the UK general adult population using standard gamble methods, and utility using this instrument can range between 0.33 and 1 [20]. The CHU-9D was completed by parental/carer as proxy for all participants aged 2–12 years only because in this trial, it was parents who consented to participate and the intervention itself is aimed at the parent/guardian as a means to improve their child’s eczema. Therefore, the study team had no contact with the child so could not ask children old enough to self-complete the CHU-9D. However, it is possible that parents/carers may have discussed it with their child when completing it.

The EQ-5D-5L is a generic preference-based instrument used to measure health-related Quality of Life. It measures health status across five dimensions (mobility, self-care, usual activities, pain/discomfort, anxiety/depression), and 5-response levels (no problems, slight problems, moderate problems, severe problems, and extreme problems) [21]. Responses received to the EQ-5D-5L were converted to utility scores, range -0.594 to 1, using the EQ-5D-5L crosswalk UK preference weights in line with recommendations at the time analysis started [22, 23]. The adult version of the EQ-5D-5L was used for all participants as the age range in the trial included both 12–15-year-olds and 16–25-year-olds, this approach is recommended in the EuroQol user guide for the EQ-5D-Y version 2 published September 2020 (https://euroqol.org/publications/user-guides/, last accessed 17 March 2023). The EQ-5D-5L has been shown to have good psychometric properties for eczema in adults [24].

The utility values were used to estimate QALYs over the trial period of 12 months, using both linear interpolation and area under the curve analysis with and without baseline adjustment [25]. Separate cost–utility analyses report the incremental cost per QALY for Trial 1 and Trial 2.

#### Patient-oriented eczema measure (POEM)

The primary outcome in both trials was the difference in eczema severity between groups as measured by the POEM at 24 weeks, with repeated measures to 12 months as a secondary outcome [26, 27]. POEM consists of 7 questions about the frequency of eczema symptoms over the previous week which when summed give a score from 0 (no eczema) to 28 (worst possible eczema) [27]. A secondary cost-effectiveness analysis was undertaken to estimate the incremental cost per success, defined as achieving at least a 2-point change on the POEM compared to baseline. A two-point change represents the smallest change on the POEM that a person would deem to be clinically important [28]. This was conducted for each trial separately and combined given the common outcome measure.

### Economic analysis

As the time horizon is 12 months in all analyses, costs and benefits were not discounted.

Treating the two trials as separate analyses, the primary analysis was an adjusted complete case cost–utility analysis where only participants with complete cost and outcome data were included in the analysis. This was chosen as the primary analysis to be in keeping with the approach taken in the analysis of the primary outcome in the statistical analysis plan. Cost and outcome data were combined for each trial to estimate an incremental cost-effectiveness ratio (ICER) comparing the online intervention plus usual care to usual care alone where appropriate. A regression-based approach (seemingly unrelated regression equations) was used in the base case cost–utility [29] and Generalized Linear Models for secondary cost-effectiveness analyses given the binary outcome.

Both unadjusted and adjusted results are presented, the latter representing the base case. The adjusted analyses adjust for baseline POEM/utility/cost (as appropriate), recruitment region and the following covariates which were pre-specified in the statistical analysis plan as possible confounders: age, gender, ethnicity, prior belief in the intervention, carer education for Trial 1, and prior use of a website or app for information or advice about the child/young person’s eczema.

Non-parametric bootstrapping was used to determine the level of sampling uncertainty surrounding the mean ICERs by generating 10,000 estimates of incremental costs and benefits. These estimates were used to produce Cost-Effectiveness Acceptability Curves to show the probability of each intervention being cost-effective at different levels of willingness to pay per QALY.

### Sensitivity analysis

Sensitivity analysis was undertaken to explore the impact of missing data comparing a complete case analysis (CCA) to multiple imputation (MI) analysis using a chained equations approach [30] with a model including the same covariates as the primary analysis. This was conducted for all participants (SA1a – unadjusted and SA2a—adjusted) and separately for those aged 2 or over (SA1b and SA2b) for trial 1.

### Subgroup analyses

No pre-specified subgroup analyses were performed because the clinical analyses found similar benefit in eczema outcomes regardless of age, gender, eczema severity, baseline treatment use, prior belief in effectiveness of intervention, or prior use of other relevant websites.

### Patient and public involvement (PPI)

Two PPI members (AR and AA) were part of the study team and contributed to aspects from the intervention development, trial design (including economic components), ongoing management meetings, through to developing/co-authoring outputs from the trial.

All analyses were undertaken in Stata 17.

## Results

### Participant characteristics

Trial 1 had 340 participants: 171 randomized to the intervention and 169 to usual care. Trial 2 had 337 participants: 168 randomized to the intervention and 169 to the usual care group. The groups were balanced in terms of demographic characteristics (see Table S1).

We first present descriptively the unadjusted mean costs and outcomes using available case data before presenting the incremental analyses.

### Intervention costs

The mean per-participant intervention cost was £1.32 in Trial 1 and £1.36 in Trial 2 over the length of the trial (see Table 3). Further details on intervention costs are provided in Supplementary Table 2.

### Wider NHS resource use and costs

Unit costs for all resources can be seen in Table 1. Eczema-related wider NHS resource use and cost (see Tables S3 and S4) were similar with no significant difference at baseline between the two groups in either trial. In trial 1, all intervention participants had complete resource use data while 167 out of 169 participants had complete resource use data in the usual care group. Trial 2 there was complete resource use data available for 166 out of 168 intervention participants and 168 out of 169 usual care group participants. The largest mean NHS resource item was medication in both trials (Table 2). The mean cost per participant by treatment group per trial is given in Table 3. In Trial 1, the mean (sd) cost per intervention participant was £138.86 (203.46), compared with a mean per-participant cost in usual care of £159.86 (231.03), giving an unadjusted mean difference of -£20.99 (95% CI  – 67.49 to 25.49) which indicates the intervention was cost saving. In Trial 2, the mean cost per intervention participant was £107.05 (sd 204.94), compared with a mean per-participant cost for usual care of £120.12 (sd 219.41) resulting in an unadjusted mean difference of -£13.08 (95% CI -58.79 to 32.64) per participant.

**Table 1:** Unit costs

Resource items	Unit cost (£ 2021)	Source
**Trial 1 and Trial 2**		
*Prescriptions*		
Medications	£0.85-£1,264.89	PCA [18]
*Primary care*		
GP/GPwSI face-to-face appointment	39.23	PSSRU [17]
GP telephone/ video/ e-consultation	50.72	
GP out of hours/walk-In center	18.11	PSSRU [31] Inflated rate from unit cost of health and social care 2016
Practice nurse face-to-face appointment	22.00	PSSRU [17]
Practice nurse telephone/ video/ e-consultation	50.72	
Practice nurse out of hours/walk-In center	35.44	PSSRU [31] Inflated rate from unit cost of health and social care 2016
Health visitor (£76), Paramedic (£89.59), and Pharmacist (£54)	59.93	PSSRU [17], Cost of a health visitor inflated from [32]
*Secondary Care*		
Referral	0.75	NHS England [19]
**Trial 1 only: Parent/carer**		
Paediatrics first appointment	133.05	NHS England [19]
Allergy first visit	272.02	NHS England [19]
Allergy clinic follow-up	344.94	NHS England [19]
Community health dermatology	86.00	NHS England [19]
Community health dermatology do not attend (DNA)	86.00	NHS England [19]
Accident and Emergency (A&E)	45.00	NHS England [19]
Phototherapy or Photochemotherapy	100.05	NHS England [19]
Dietician	92.00	NHS England [19]
NHS111 call	13.37	Pope C et al. [33]
Paediatric assessment unit	220.52	Jones R. [34]
**Trial 2 only: Young person**		
Outpatient dermatology first appointment (Non-Admitted Face-to-Face Attendance)	156.46	NHS England [19]
Outpatient dermatology follow-up appointment (Non-Admitted Face-to-Face Attendance)	149.15	NHS England [19]
Outpatient dermatology first appointment (Non-admitted non-Face to Face)	170.89	NHS England [19]
Outpatient dermatology follow-up appointment (Non-admitted non-Face to Face)	119.45	NHS England [19]
Phototherapy or Photo-chemotherapy	257.00	NHS England [19]
Remote dermatology appointment (Telephone or online)	170.89	NHS England [19]
Dermatology nurse advanced practitioner	203.99	NHS England [19]

**Table 2:** Mean (SD) and Mean difference (95% CI) resource use for both Trial 1 and Trial 2 by intervention group over 12 months (based on available data)

*Trial 1 (Parent/Carer)*					
	Online intervention (*n* = 171/171)		Usual Care (*n* = 167/169)		Mean difference
	Mean	Std dev	Mean	Std dev	(95% CI)
Medication prescriptions (Number of items)	5.16	8.11	5.76	9.51	– 0.60 ( – 2.49 to 1.28)
**Primary care consultation (number of visits)**					
GP visits	0.87	1.63	0.77	1.43	0.09 ( – 0.24 to 0.42)
Practice nurse	0.04	0.29	0.00	0.00	0.04 ( – 0.01 to 0.08)
Nurse practitioner	0.15	0.49	0.13	0.50	0.02 ( – 0.09 to 0.12)
Health visitor, Paramedic, Pharmacist, others	0.12	0.45	0.13	0.48	– 0.01 ( – 0.11 to 0.09)
**Secondary care Consultations (number of visits)**	0.29	0.83	0.57	1.46	– 0.27 ( – 0.53 to 0.02)

*Trial 2 (Young Person)*					
	Online intervention (*n* = 166/168)		Usual Care (*n* = 168/169)		
	Mean	Std dev	Mean	Std dev	(95% CI)
Medication prescriptions (Number of items)	3.52	5.74	4.47	7.80	– 0.95 ( – 2.43 to 0.52)
**Primary Care Consultation (number of visits)**					
GP visits	0.52	1.26	0.76	1.46	– 0.24 ( – 0.53 to 0.06)
Practice nurse	0 .02	0.13	0 .05	0 .33	– 0.04 ( – 0.09 to 0.02)
Nurse practitioner	0.13	0.46	0.17	0.67	– 0.04 ( – 0.16 to 0.08)
Health visitor, Paramedic, Pharmacist, others	0 .07	0.30	0 .08	0.34	– 0.01 ( – 0.08 to 0.06)
**Secondary care Consultations (number of visits)**	0.31	0 .98	0.38	1.36	– 0.07 ( – 0.33 to 0.18)

**Table 3:** Mean (sd) and mean difference (95% CI) costs for both Trial 1 and Trial 2 by intervention group over 12 months (UK £2021 Sterling) (based on available data)

*Trial 1 (Parent/Carer)*					
	Online intervention (*n* = 171/171)		Usual care (*n* = 167/169)		Mean difference
	Mean	Std dev	Mean	Std dev	95% CI
Intervention maintenance and ongoing delivery costs	1.32	0.00	0.00	0.00	1.36 (1.32 to 1.32)
Medication prescription	35.22	68.9	41.72	93.42	– 6.50 ( – 24.03 to 11.02)
GP visits	42.97	83.05	37.36	68.74	5.61 ( – 10.69 to 21.91)
Practice nurse	1.78	14.45	0.00	0.00	1.78 ( – 0.41 to 3.97)
Nurse practitioner	7.71	25.82	5.49	20.75	2.23 ( – 2.78 to 7.24)
Others (inc Health visitor, Paramedic, Pharmacist)	7.05	27.52	7.54	29.55	– 0.49 ( – 6.60 to 5.63)
Secondary care Consultations	44.17	154.95	67.8	199.31	– 23.63 ( – 61.73 to 14.47)
**Total Healthcare cost**	**138.86**	**203.46**	**159.86**	**231.03**	** – 20.99 ( – 67.49 to 25.49)**

*Trial 2 (Young Person)*					
	Online Intervention (*n* = 166/168)		Usual Care (*n* = 168/169)		Mean difference
	Mean	Std dev	Mean	Std dev	95% CI
Intervention maintenance and ongoing delivery costs	1.36	0.00	0.00	0.00	1.36 (1.36 to 1.36)
Medication Prescription (With dupilumab injection)	33.11	109.74	27.93	49.17	5.18 ( – 13.09 to 23.45)
Medication Prescription (Without dupilumab injection)	25.65	53.00	27.93	49.17	– 2.29 ( – 13.34 to 8.73)
GP visits	25.15	59.18	36.2	69.63	– 11.05 ( – 24.97 to 2.87)
Practice Nurse	0.92	6.78	2.38	15.64	– 1.46 ( – 4.06 to 1.14)
Nurse Practitioner	6.38	22.16	8.45	31.23	– 2.08 ( – 7.91 to 3.76)
Others (inc Health visitor, Paramedic, Pharmacist)	4.33	18.15	4.64	18.55	– 0.31 ( – 4.26 to 3.65)
Secondary care Consultations	35.8	129.33	40.53	152.54	– 4.73 ( – 35.19 to 25.72)
**Total Healthcare cost**	**107.05**	**204.94**	**120.12**	**219.41**	** – 13.08 ( – 58.79 to 32.64)**

## Outcomes

Mean outcomes are presented in Table 4.

**Table 4:** Mean (SD) and mean difference (95% CI) in outcomes at each time point for Trial 1 and Trial 2 by intervention arm over 12 months (available case data)

	Online Intervention (*n* = 171)		Usual Care (*n* = 169)		
	Mean	Std dev (*n*)	Mean	Std dev (*n*)	Mean Difference (95% CI)
*Trial 1 (Parent/Carer*)					
**CHU-9D**					
Baseline CHU-9D	0.868	0.093(140)	0.858	0.094 (132)	0.01 ( – 0.012 to 0.032)
24 weeks CHU-9D	0.894	0.087 (96)	0.877	0.109 (106)	0.017 ( – 0.01 to 0.045)
52 weeks CHU-9D	0.901	0.089 (119)	0.881	0.103 (121)	0.02 ( – 0.004 to 0.045)
QALYs at 52 weeks	0.890	0.075 (89)	0.877	0.085 (96)	0.014 ( – 0.01 to 0.037)
**POEM Scores**					
Baseline POEM	12.877	5.17 (171)	12.713	5.346(167)	0.165 ( – 0.961 to 1.29)
24 weeks POEM	9.078	6.201 (153)	10.752	6.334 (157)	– 1.673 ( – 3.074 to – 0.272)
52 weeks POEM	8.926	6.727 (149)	9.941	6.549 (153)	– 1.015 (2.518 to 0.488)
Change in POEM	-3.617	6.416 (149)	– 2.719	5.521 (153)	– 0.898 ( – 2.253 to 0.456)

	% (number)	Std Err	% (number)	Std Err	Difference in proportions (95% CI)
Proportion achieving success at 52 weeks	65.10 (97)	3.91 (149)	57.79 (89)	3.98 (154)	7.31 ( – 3.7 to 18.3)

	Online Intervention (*n* = 168)		Usual Care (*n* = 169)		
	% (number)	Std Err	% (number)	Std Err	Difference in proportions (95% CI)
*Trial 2 (Young Person)*					
**EQ-5D-5L**					
Baseline EQ-5D-5L	0.801	0.145 (168)	0.798	0.175 (169)	0.004 ( – 0.031 to 0.038)
24 weeks EQ-5D-5L	0.803	0.180 (138)	0.795	0.183 (154)	0.008 ( – 0.034 to 0.050)
52 weeks EQ-5D-5L	0.826	0.166 (133)	0.794	0.166 (147)	0.032 ( – 0.007 to 0.071)
QALYs at 52 weeks	0.813	0.138 (123)	0.803	0.138 (140)	0.010 ( – 0.024 to 0.043)
**POEM Scores**					
Baseline POEM	15.079	5.279 (165)	15.27	5.503 (169)	– 0.199 ( – 0.962 to 1.361)
24 weeks POEM	11.621	6.647 (140)	13.826	7.110 (161)	– 2.204 ( – 3.761 to – 0.647)
52 weeks POEM	10.598	6.517 (132)	12.696	6.839 (148)	– 2.097 ( – 3.674 to – 0.533)
Change in POEM	4.341	6.825 (132)	2.351	6.930 (148)	1.989 (0.368 to 3.611)

	% (number)	Std Err	% (number)	Std Err	Difference in proportions (95% CI)
Proportion achieving success at 52 weeks	66.7 (88)	4.10 (132)	54.7 (81)	4.09 (148)	11.9 ( – 0.58 to 23.29)

### Quality-adjusted life years (QALYS)

In Trial 1, complete responses to the CHU-9D were available for 96% at baseline, 72% at 6 months and 85% at 12 months, with 34% missing overall. There were 58 (17%) children aged under 2 whose parents/carers were not asked the CHU-9D and thus excluded from the primary analysis. Of those completing the CHU-9D at baseline 11% in the intervention group and 7% in the usual care group reported being in perfect health at baseline. Thsis percentage increased to 20% and 17% respectively by 52 weeks. The mean (sd) QALYs for the intervention group after 12 months was 0.891 (0.075) per participant compared to 0.877 (0.085) per participant for usual care, resulting in a mean difference of 0.014 (95% CI  – 0.010 to 0.037) QALYs.

In Trial 2, complete responses for the EQ-5D-5L were available for 100% at baseline, 87% at 6 months, and 83% at 12 months, with a total of 22% missing overall. Of those completing the EQ-5D-5L at baseline, 19% in the intervention group and 21% in the usual care group reported being in perfect health at baseline. This percentage increased to 30% and 21% respectively by 52 weeks. The mean (sd) per-participant QALYs in the intervention group was 0.813 (0.138) compared to 0.803 (0.138) in the usual care group. This resulted in an incremental mean difference in QALYs of 0.010 (95% CI -0.024 to 0.043) over the 12-month trial period.

### Patient-oriented eczema measure (POEM)

In Trial 1, the proportion achieving success (Standard Error, SE), defined as at least a 2-point change in POEM, was 65.1% (3.91) for the intervention group compared to 57.8% (3.98) for usual care, such that the difference in proportions was 7.31% (95% CI -3.7 to 18.3) (10.8% responses missing at 12 months). For Trial 2, the corresponding figures were 66.7% (4.10) compared to 54.7% (4.09), with a difference of proportions of 11.9% (95% CI  – 0.58 to 23.3) (17% of responses missing).

### Primary analysis: cost–utility

The incremental analysis results for both trials are presented in Table 5.

**Table 5:** Incremental cost–utility analyses and cost-effectiveness analyses results, including Sensitivity Analyses (SA) for both Trial 1 and Trial 2

*Trial 1 (Parent/Carer)*					
Analysis (*n*_*i*_, *n*_uc_) (171,169)	Incremental Cost (£) (95% CI)	Incremental QALYs (95% CI)	ICER (£)	CEAC at £20,000 threshold	CEAC at £30,000 threshold
Base-case, CCA, Unadjusted (89, 96)	3.08 ( – 59.20 to 65.36)	0.014 ( – 0.009 to 0.037)	227.49	87%	87%
Base-case, CCA, Adjusted (73, 71)	– 34.15 ( – 104.54 to 36.24)	-0.003 ( – 0.021 to 0.015)	12,465.86	69%	68%
SA1a, MI for all participants, Unadjusted (171,169)	– 21.03 ( – 67.24 to 25.18)	0.016 ( – 0.003 to 0.035)	Dominant	87%	87%
SA2a, M1 for all participants, Adjusted (171,169)	– 23.60 ( – 68.59 to 21.40)	0.007 ( – 0.007 to 0.021)	Dominant	65%	63%
SA1b, MI for children aged 2 and over only, Unadjusted (142, 140)	– 6.73 ( – 55.4 to 41.98)	0.017 ( – 0.003 to 0.036)	Dominant	87%	87%
SA2b, MI for children aged 2 and over only, Adjusted (142, 140)	– 21.62 ( – 68.78 to 25.55)	0.011 ( – 0.003 to 0.025)	Dominant	69%	68%

Analysis (n_i_, n_uc_) (168, 169)	Incremental Costs (95% CI)	Incremental Proportion achieving success (95% CI)	ICER		
Secondary analysis, CEA, Unadjusted (149,153)	– 22.88 ( – 72.27 to 26.52)	7.6% ( – 3.4% to 18.6%)	Dominant		
Secondary analysis, CEA, Adjusted (97, 88)	– 27.66 ( – 79.63 to 24.31)	8.6% ( – 3.0% to 20.2%)	Dominant		

Trial 2 (Young Person)					
Analysis (n_i_, n_uc_)	Incremental Cost (£) (95% CI)	Incremental QALYs (95% CI)	ICER	CEAC at £20,000 threshold	CEAC at £30,000 threshold
Base-case, CCA, Unadjusted (122,140)	– 25.56 ( – 74.68 to 23.56)	0.010 ( – 0.023 to 0.044)	Dominant	75%	74%
Base-case, CCA, Adjusted (88,118)	– 20.82 ( – 71.77 to 30.13)	0.012 ( – 0.017 to 0.041)	Dominant	81%	81%
SA1a, MI Unadjusted (168,169)	– 13.66 ( – 59.05 to 31.73)	0.016 ( – 0.017 to 0.476)	Dominant	84%	83%
SA2a, M1 Adjusted (168,169)	– 11.77 ( – 54.27 to 230.71)	0.008 ( – 0.015 to 0.031)	Dominant	81%	80%

Analysis (n_i_, n_uc_)	Incremental Costs (95% CI)	Incremental Proportion achieving success (95% CI)	ICER		
Secondary analysis, CEA, Unadjusted (131,147)	– 19.24 ( – 68.50 to 30.02)	11.3% ( – 0.2% to 22.8%)	Dominant		
Secondary analysis, CEA, Adjusted (96,123)	– 23.57 ( – 74.22 to 23.07)	10.4% ( – 2.4% to 23.2%)	Dominant		

Combined CEA analysis for trials 1 and 2					
Analysis (n_i_, n_uc_)	Incremental Costs (95% CI)	Incremental proportion achieving success (95% CI)	ICER		
Secondary analysis, CEA, Unadjusted (280,300)	– 20.36 ( – 55.38 to 16.66)	9.4% (1.4% to 17.3%)	Dominant		
Secondary analysis, CEA, Adjusted (270,289)	– 20.35 ( – 55.41 to 14.70)	10.3% (2.3% to 18.1%)	Dominant		

*For Incremental Proportion achieving success (> 2-point change on POEM) adjusted analysis, ‘Prior belief in effectiveness of website’ was removed from the analysis due to the model being unable to converge if it was included. Where n_i_ is the number of participants with data available in the intervention group; n_uc_ the number of participants in the usual care group with data available; CUA is cost–utility analysis; CEA is cost-effectiveness analysis; CCA is cost consequence analysis and MI is multiple imputation

In Trial 1 (parent/carer), complete case analysis, the adjusted incremental cost was  – £34.15 (95% CI  – 104.54 to 36.24) per participant for the intervention group (*n* = 89) compared to the usual care group (*n* = 96). Thus, the intervention was cost saving with small incremental QALYs of  – 0.003 (95% CI  – 0.021 to 0.015) very slightly in favor of the usual care group. The estimated ICER was £12,465.86 per QALY. The probability that the intervention is cost-effective was 69% and 68% at the £20,000 and £30,000 willingness to pay thresholds respectively (see Figure S1) suggesting a 31(32)% chance of decision-makers reaching a wrong decision should Trial 1 intervention be offered in addition to usual eczema care in the NHS.

In Trial 2, the adjusted incremental cost was -£20.82 (95% CI -71.77 to 30.13) for the intervention group (*n* = 86) compared to the usual care group (*n* = 118) and was associated with incremental QALYs of 0.012 (95% CI  – 0.017 to 0.041). Since the intervention dominated usual care, an ICER was not calculated. The probability that the intervention was cost-effective was 81% at both the £20,000 and £30,000 willingness to pay threshold (see Figure S1) suggesting a 19% chance of decision-makers reaching a wrong decision should Trial 2 intervention be offered in addition to usual eczema care in the NHS.

### Secondary analysis: cost-effectiveness

In all cost-effectiveness analyses, the intervention is cheaper and more effective than usual care. In the complete case analysis for Trial 1, 97 out of 149 participants in the intervention group and 88 out of 153 participants in the usual care group had both complete cost and POEM data. The adjusted incremental cost difference of  – £27.66 (95% CI  – 79.63 to 24.31) was associated with an incremental difference in terms of proportion success of 8.6% (95% CI  – 3.0 to 20.2).

In the adjusted complete case analysis for Trial 2, 96 out of 131 participants in the intervention group and 123 out of 147 participants in the usual care group had both complete cost and POEM scores data. The adjusted incremental cost difference of  – £23.57 (95% CI  – 74.22 to 23.07) was associated with an adjusted incremental difference in terms of proportion success of 10.4% (95% CI  – 2.4 to 23.2).

The online digital behavior interventions remained dominant when the population from both trials was combined with an adjusted incremental cost of  – £20.35 (95% CI  – 55.41 to 14.70) associated with an incremental difference in terms of proportion success of 10.3% (95% CI 2.3 to 18.1).

### Sensitivity analyses

In the sensitivity analysis, multiple imputation was used to explore the impact of missing data on results. 46% (156) and 22% (75) of participants had data missing (costs and/or utility) in Trials 1 and 2 respectively. For Trial 1, undertaking multiple imputation for all participants resulted in the intervention becoming dominant with an incremental cost of  – £23.60 (95% CI  – 68.59 to 21.40), and incremental QALY of 0.007 (95% CI  – 0.007 to 0.021). When this analysis was run just for those participants aged 2 years and over (SA1b and SA2b) (whose parents were asked the CHU-9D, and therefore had difficulties or choose not to respond if it was missing), the results were similar, and the conclusion reached the same. In Trial 2, the results did not change from the primary analysis such that the intervention remained dominant with an incremental cost of  – £11.77 (95% CI  – 54.27 to 230.71) and incremental QALYs of 0.008 (95% CI  – 0.015 to 0.031).

## Discussion

This study finds that EczemaCareOnline.org.uk, which supports eczema self-care management for both parent/carers and young people, is both low cost to maintain and highly cost-effective compared to usual care. In the majority of analyses, the online intervention was estimated to be dominant (less costly and more effective) over usual care alone. The two exceptions were in Trial 1, where in the cost–utility complete case analysis, the incremental cost-effectiveness ratios were estimated as £227.49 and £12,466 per QALY for the unadjusted and adjusted analyses respectively. Estimated differences in QALYs were very small. In addition, the complete case analysis excluded 17% of the sample because we did not ask carers to complete the CHU-9D if their child was aged under 2 at time of recruitment due to the instrument not being appropriate for this age range.

A priori it was unclear whether the interventions were likely to increase or reduce overall costs and how costs would differ for different types of resources within the overall picture. For instance, it was conceivable that providing better-quality self-management support could lead to increased medication costs as people may be more likely to request and use more appropriate items to manage their condition while at the same time, this might lead to less secondary care costs if less referrals are needed due to better management of the eczema. The results from the trials show us that where cost savings were achieved, this differed slightly between trials, and in Trial 1, secondary care appointments, medications, and others (including pharmacist and health visitor) saw lower costs in the intervention arm compared to usual care, whereas in Trial 2, all categories except medications saw lower costs in the intervention arm compared to usual care. Medication costs were higher in Trial 2 due to one participant having dupilumab injection (approximately 12 times the price of the next most expensive capsorin medication for a participant in the usual care group). Despite all costs being small, they suggest that secondary care costs may be reduced by greater use of medications by those with eczema and this would be worthy of further research.

Sensitivity analyses supported the conclusion that costs were reduced and outcomes improved using the intervention. There was little uncertainty associated with the decision to adopt the online behavior intervention as part of regular care for people with eczema in the UK. This was so over a 12-month period which suggests the interventions achieve good value for money over a relatively short space of time.

Compared to the cost of delivering the nurse-led ‘Eczema Education Programme’ (EEP), for parents of children with eczema (£120 per family)[5], it can be seen that ECO (£1.32/£1.36 per family) has potential to be significantly cheaper as it can reach many more families. This finding is in keeping with that of different online interventions for other conditions where economic evaluations have similarly found them to be cost-effective [35, 36]. For instance, a web-based self-management intervention for patients with type 2 diabetes (HeLP-Diabetes) compared to usual care in the UK was cost-effective at £5,550 per QALY [35]. It is, however, possible the ongoing costs of maintaining the online intervention (including updating the intervention) will be higher outside of a trial environment, but many more individuals will be able to access the intervention, so ongoing costs per head are likely to be minimal.

It is a strength that this study was conducted alongside a clinical trial which enabled the collection and analysis of individual participant level data over a 12-month period and is a widely accepted method of measuring the effectiveness of healthcare interventions [9]. However, we did not collect the CHU-9D for participants aged under 2 years as the instrument is not available in a format for this age group. Thus, the complete case cost–utility analyses had to exclude these participants. It was also not possible to take a broader perspective to collect participant costs given the study design. The trials were set up to have minimal contact with the participants to reflect how the interventions would be used in practice. Therefore, only outcome data were collected from participants at baseline, 24 and 52 weeks. Further research to explore the cost implications of digital type interventions for participants, both in terms of the time to use the intervention and the knock-on impacts on out-of-pocket expenses related to the condition of interest, would be useful.

Further in common with studies of this type, the study did experience some missing data, mostly for outcomes. However, sensitivity analysis was undertaken to explore the impact of missing data, by comparing the complete case analyses to multiple imputation. In Trial 1, taking account of the missing data shifted the online intervention to dominate usual care.

In common with other eczema trials, it was observed that outcomes improved even in the usual care groups in this trial. This may reflect that the study collected data on symptoms regularly and by doing this encouraged participants to think more about their eczema, which may in turn encourage them to look after their eczema more than had they not entered the study. This could have impacted on both groups and as such it is not clear what if any effect this would have on the differences in outcomes observed between groups.

Our research involved the development of an implementation plan for if the interventions were found effective and cost-effective which can now be actioned. Further research could explore the appropriateness of EczemaCareOnline.org.uk for other healthcare systems.

To conclude, the online self-management interventions for eczema are freely available on a single website (EczemaCareOnline.org.uk) to ease uptake. This study found the interventions to be low cost to maintain and cost-effective.

## Supplementary Information

Below is the link to the electronic supplementary material.

Supplementary file1 (DOCX 68 KB)

Supplementary file2 (DOCX 210 KB)

Supplementary file3 (DOCX 24 KB)

## Data Availability

Consent was not obtained from participants for data sharing. Authors will consider reasonable request to make relevant anonymized participant level data available.
