# Supplementary material for: Cost-effectiveness of two online interventions supporting self-care for eczema for parents/carers and young people
Source: Eur J Health Econ. 2024 Jan 9;25(7):1165–76. doi: 10.1007/s10198-023-01649-9 (PMC11377600; doi:10.1007/s10198-023-01649-9)

**Supporting Information for:**

**Title:**

Cost effectiveness of two online interventions supporting self-care for eczema for parents/carers and young people

*Running Head:* Cost effectiveness of two online self-care interventions for eczema

**Appendix S1 Supplementary material**

**Supplementary Table 1 (Table S1); Participant characteristics (breakdown presented in supplementary material TS1 for both trials)**

|  | Trial 1 (Parent/Carer) | | | | Trial 2 (Young person) | |
| --- | --- | --- | --- | --- | --- | --- |
| Characteristics | Online Intervention (n=171) | | Usual Care (n=169) | | Online Intervention (n=168) | Usual Care (n=169) |
| Age, mean (SD) | 4.75 (3.31) (n=171) | | 4.79 (3.35) (n=169) | | 19.46 (3.49)  (n= 168) | 19.04 (3.33)  (n= 169) |
| Parent/Carer Age, mean (SD) | 37.70 (6.83) (n=171) | | 37.52 (6.39) (n=169) | |  |  |
| Female, number (%) | 85 (50%)  (n= 171) | | 79 (47%)  (n= 169) | | 125 (74%)  (n= 168) | 134 (79%)  (n= 169) |
| Parent/Carer Female, number (%) | 156 (91%)  (n= 171) | | 155 (92%)  (n= 169) | |  |  |
| Ethnic group, number (%) | n= 169 | n= 166 | | n= 164 | | n= 166 |
| White | 144 (84%) | | 138 (82%) | | 142 (87%) | 142 (86%) |
| African | 2 (1%) | | 7 (4%) | | 3 (2%) | 2 (1%) |
| Mixed | 7 (4%) | | 6 (4%) | | 9 (6%) | 10 (6%) |
| Asian | 10 (6%) | | 13 (8%) | | 7 (4%) | 9 (5%) |
| Other | 6 (6%) | | 2 (1%) | | 3 (2%) | 3 (2%) |
| Centre, number (%) | n= 172 | n= 169 | | n= 168 | | n= 169 |
| 1 | 52 (31%) | | 58 (34%) | | 44 (26%) | 46 (27%) |
| 2 | 36 (21%) | | 29 (17%) | | 43 (26%) | 44 (26%) |
| 3 | 50 (29%) | | 38 (23%) | | 47 (28%) | 45 (27%) |
| 4 | 33 (19%) | | 44 (26%) | | 34 (20%) | 34 (20%) |
| Prior believe, mean (SD) | 6.73 (2.12) (n=142) | | 6.95 (2.16) (n=132) | | 6.29 (2.17)  (n= 123) | 6.36 (1.91)  (n= 139) |
| Prior use of website or app, number (%) | 41 (24%)  (n= 171) | | 31 (18%)  (n= 163) | | 26 (16%)  (n= 162) | 24 (14%)  (n= 167) |
| Highest education level of parent, number (%) | n=168 | n=164 | | |  | |
| Degree | 80 (47%) | | 87 (52%) | |  |  |
| Diploma | 29 (17%) | | 22 (13%) | |  |  |
| A-level | 6 (4%) | | 10 (6%) | |  |  |
| GCSEs/O level | 19 (11%) | | 14 (8%) | |  |  |
| Other | 23 (13%) | | 24 (14%) | |  |  |
| None | 5 (3%) | | 3 (2%) | |  |  |
| Prefer not to say | 6 (5%) | | 4 (5%) | |  |  |

**Intervention costs**

Supplementary Table 2 provides a breakdown of the intervention development costs and ongoing intervention costs as delivered in the trial. Total intervention development cost £63,542 per trial intervention. The total ongoing intervention cost for each of the trials was £226. If implemented the ongoing costs may differ to those incurred during the trial.

**Supplementary Table 2 (Table S2): Total development and ongoing intervention cost for each of the trials 1 and 2**

| **Description** | | **Trial 1 (Parent/Carer)** | **Trial 2**  **(Young Person)** |
| --- | --- | --- | --- |
| **Intervention development** | | **£63,542** | **£63,542** |
| Research fellows | 1 FTE research fellow for 24 months per intervention | £35,000 | £35,000 |
| Video development | £1000 per video x 4 | £2000 | £2,000 |
| Intervention development group (expert panel, including PPI) | Expert panel of 14 members (2 dermatologists, 3 GPs, 1 nurse consultant, 1 Prof of nursing, 2 x academic skin researchers, 2 psychologists, 3 PPI). Each group member gave approx. 15 hours.  Cost calculated based on NIHR recommended rates | £8,280 | £8,280 |
| Life-Guide server | Interventions and trials were delivered using Life-Guide. | £250 | £250 |
| Life-Guide technical support | Level 4 Research Fellow, 1 day/week 2 yrs. 5 day/week 3 years | £15,000 | £15,000 |
| Software developer (for dissemination) | Commercial software developer to move the intervention onto a different platform for dissemination. We are paying a fixed fee. | £34,000 | £34,000 |
| SMS costs | Text messaging during the trial – we bought 30,000 text bundle | £512 | £512 |
| **Ongoing costs** | | **£226** | **£226** |
| Server to host intervention |  | £150 | £150 |
| Emails | This will be free for up to a point and then £11 a month (for both interventions) | £66 | £66 |
| Domain name |  | £10 | £10 |

**Supplementary Table 3 (Table S3): Mean (sd) and mean difference (95% CI) baseline resource use for both trial 1 and trial 2 (based on available data)**

| **Trial 1 (Parent/Carer)** | | | | | |
| --- | --- | --- | --- | --- | --- |
| **Item (Ni, Nc)** | **Online intervention (n=171/171)** | | **Usual Care**  **(n=167/169)** | | **Mean difference** |
|  | **Mean** | **Std dev** | **Mean** | **Std dev** | **(95% CI)** |
| Medication Prescriptions | 1.57 | 2.30 | 1.66 | 3.10 | -0.09 (-0.68 to 0.49) |
| **Primary Care Consultation** | | | | | |
| GP visits | 0.36 | 0.75 | 0.35 | 0.86 | 0.01 (-0.16 to 0.18) |
| Practice Nurse | 0.01 | 0.11 | 0.05 | 0.41 | -0.04 (-0.10 to 0.03) |
| Nurse Practitioner | 0.12 | 0.37 | 0.06 | 0.24 | 0.06 (-0.01 to 0.12) |
| Health visitor, Paramedic, Pharmacist, others | 0.05 | 0.22 | 0.03 | 0.20 | 0.02 (-0.02 to 0.07) |
| **Secondary care Consultations** | 0.22 | 0.82 | 0.24 | 1.02 | -0.02 (-0.22 to 0.18) |
| **Trial 2 (Young Person)** | | | | | |
|  | **Online intervention (n=166/168)** | | **Usual Care**  **(n=168/169)** | | **Mean difference** |
|  | **Mean** | **Std dev** | **Mean** | **Std dev** | **(95% CI)** |
| Medication Prescriptions | 1.07 | 2.04 | 1.27 | 2.56 | -0.20 (-0.70 to 0.30) |
| **Primary Care Consultation** | | | | | |
| GP visits | 0.16 | 0.50 | 0.23 | 0.60 | -0.08 (-0.19 to 0.04) |
| Practice Nurse | 0.00 | 0.00 | 0.02 | 0.13 | -0.02 (-0.04 to 0.002) |
| Nurse Practitioner | 0 .04 | 0.19 | 0.01 | 0.11 | 0.02 (-0.01 to 0.06) |
| Health visitor, Paramedic, Pharmacist, others | 0.02 | 0.13 | 0.02 | 0.15 | -0.01 (-0.04 to 0.03) |
| **Secondary care Consultations** | 0.10 | 0.39 | 0.08 | 0.36 | 0.03 (-0.06 to 0.11) |

**Supplementary Table 4 (Table S4): Mean (sd) and mean difference (95% CI) baseline cost (UK£2021) for both trial 1 and trial 2 (based on available data)**

| **Trial 1 (Parent/Carer)** | | | | | |
| --- | --- | --- | --- | --- | --- |
|  | **Online intervention (n=171/171)** | | **Usual Care**  **(n=167/169)** | | **Mean difference** |
|  | **Mean** | **Std dev** | **Mean** | **Std dev** | **(95% CI)** |
| **Medication Prescriptions** | 11.79 | 25.28 | 10.73 | 23.90 | 1.06 (-4.20 to 6.32) |
| **Primary Care Consultation** | | | | | |
| GP visits | 18.27 | 45.44 | 18.26 | 52.06 | 0.02 (-10.42 to 10.45) |
| Practice Nurse | 0.59 | 5.47 | 2.04 | 20.02 | -1.44 (-4.57 to 1.68) |
| Nurse Practitioner | 6.28 | 23.72 | 3.21 | 13.03 | 3.06 (-1.03 to 7.16) |
| Health visitor, Paramedic, Pharmacist, others | 3.86 | 18.55 | 2.14 | 14.51 | 1.72 (-1.85 to 5.28) |
| **Secondary care Consultations** | 27.10 | 124.22 | 23.76 | 136.27 | 3.34 (-24.51 to 31.19) |
| **Total health care costs Trial 1** | **67.89** | **150.24** | **60.74** | **166.94** | **7.16 (-26.76 to 41.07)** |
| **Trial 2 (Young Person)** | | | | | |
|  | **Online intervention (n=166/168)** | | **Usual Care**  **(n=168/169)** | | **Mean difference** |
|  | **Mean** | **Std dev** | **Mean** | **Std dev** | **(95% CI)** |
| **Medication Prescriptions** | 7.12 | 16.35 | 7.55 | 15.66 | -0.44 (-3.88 to 3.01) |
| **Primary Care Consultation** | | | | | |
| **GP visits** | 7.03 | 21.80 | 10.45 | 26.36 | -3.42 (-8.63 to 1.79) |
| **Practice Nurse** | 0.00 | 0.00 | 0.91 | 6.74 | -0.91 (-1.93 to 0.12) |
| **Nurse Practitioner** | 1.49 | 8.12 | 0.60 | 5.52 | 0.88 (-0.61 to 2.38) |
| **Health visitor, Paramedic, Pharmacist, others** | 0 .72 | 6.56 | 1.43 | 9.16 | -0.71 (-2.42 to 1.01) |
| **Secondary care Consultations** | 12.52 | 58.65 | 8.28 | 41.91 | 4.25 (-6.72 to 15.21) |
| **Total health care costs Trial 2** | **28.87** | **74.96** | **29.21** | **69.94** | **-0.34 (-15.42 to 14.74)** |

**Figure S1: Cost effectiveness Acceptability Curve for the Eczema Care Online (ECO) intervention for trial 1 and Trial 2 (Adjusted, complete case, cost utility analysis)**


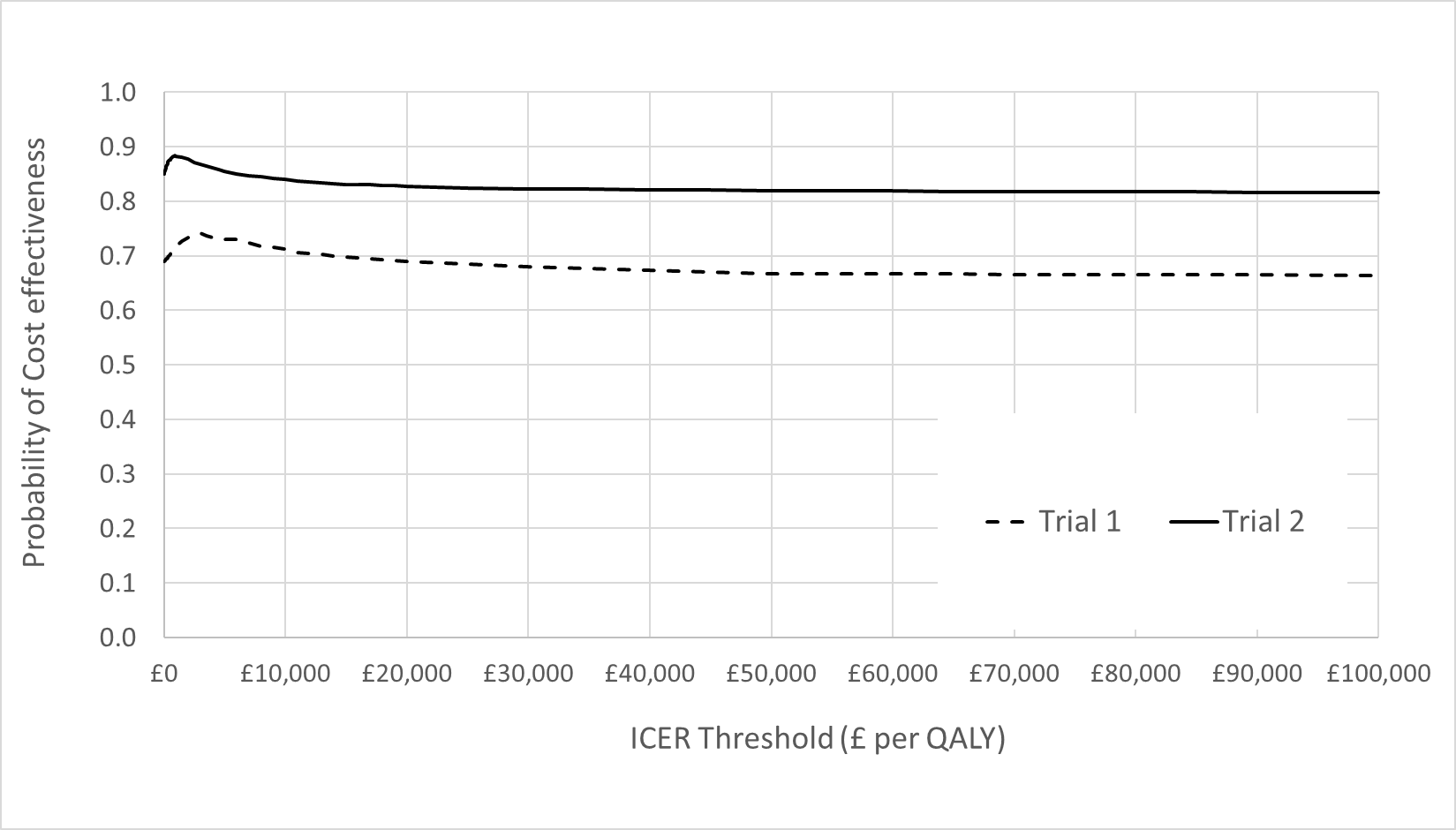

Supplement: Supplementary file 1 — Supplementary file1 (DOCX 68 KB) [file 10198_2023_1649_MOESM1_ESM.docx]
