# Supplementary material for: Cost-effectiveness of two online interventions supporting self-care for eczema for parents/carers and young people
Source: Eur J Health Econ. 2024 Jan 9;25(7):1165–76. doi: 10.1007/s10198-023-01649-9 (PMC11377600; doi:10.1007/s10198-023-01649-9)
Supplement: Supplementary file 2 — Supplementary file2 (DOCX 210 KB) [file 10198_2023_1649_MOESM2_ESM.docx]

**Supporting Information for:**

**Title:**

Cost effectiveness of two online interventions supporting self-care for eczema for parents/carers and young people

*Running Head:* Cost effectiveness of two online self-care interventions for eczema


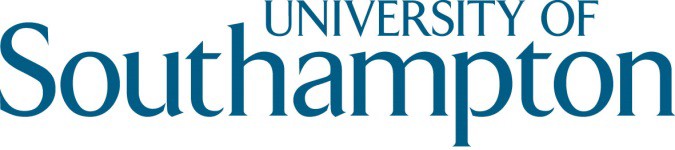


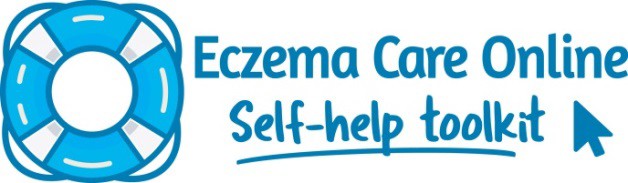


**Notes Review Form**


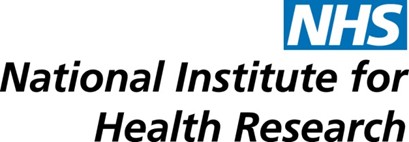


| **STUDY PARTICIPANT DETAILS:** | | |  | **YOUR DETAILS:** | |
| --- | --- | --- | --- | --- | --- |
| **STUDY ID:** |  | |  | **SURGERY NAME:** |  |
| **INITIALS** |  | |  | **YOUR NAME:** |  |
| **Patient notes review period** | **From:** | **To:** |  | **TODAY’S DATE:** |  |

# Please tick if patient has left this surgery: Date of leaving (if known): COMMENTS:

1. Please record details of **all oral or topical treatments that are likely to have been prescribed for eczema during the notes review period listed**. Include formulation (e.g. cream, ointment) and strength of topical medications. (Continue overleaf if necessary).

| **Date of prescription** | **Item prescribed**  ***(e.g. Doublebase gel, Hydrocortisone cream 1%)*** | **Amount prescribed**  ***(e.g. 550g, 100ml, 28 tablets)*** | **Was this prescription for eczema?** | | |
| --- | --- | --- | --- | --- | --- |
|  |  |  | **Yes** | **Not sure** | |
|  |  |  |  | |  |
|  |  |  |  | |  |
|  |  |  |  | |  |
|  |  |  |  | |  |
|  |  |  |  | |  |
|  |  |  |  | |  |
|  |  |  |  | |  |
|  |  |  |  | |  |
|  |  |  |  | |  |
|  |  |  |  | |  |
|  |  |  |  | |  |
|  |  |  |  | |  |

- 1. **Prescriptions (continued)**

| **Date of prescription** | **Item prescribed**  ***(e.g. Doublebase gel, Hydrocortisone cream 1%)*** | **Amount prescribed**  ***(e.g. 550g, 100ml, 28 tablets)*** | **Was this prescription for eczema?** | | |
| --- | --- | --- | --- | --- | --- |
|  |  |  | **Yes** | **Not sure** | |
|  |  |  |  | |  |
|  |  |  |  | |  |
|  |  |  |  | |  |
|  |  |  |  | |  |
|  |  |  |  | |  |
|  |  |  |  | |  |
|  |  |  |  | |  |
|  |  |  |  | |  |
|  |  |  |  | |  |
|  |  |  |  | |  |
|  |  |  |  | |  |
|  |  |  |  | |  |

- 1. **Please record ALL possible eczema related CONSULTATIONS during the date period listed (continue overleaf if necessary):**

| **Date of consultation** | **Type of Consultation** | | | | | **Health Professional seen** | | | | **Was this consultation about eczema? (tick one box)** | |
| --- | --- | --- | --- | --- | --- | --- | --- | --- | --- | --- | --- |
|  | **Did not attend booked appointment (DNA)** | **Face to face appointment** | **Telephone/ video / e-consultation** | **Home visit** | **Out of Hours / Walk-In Centre** | **GP** | **Practice Nurse** | **Nurse Practitioner** | **Health Visitor, Paramedic, Pharmacist or other** | **Yes** | **Not Sure** |
|  |  |  |  |  |  |  |  |  |  |  |  |
|  |  |  |  |  |  |  |  |  |  |  |  |
|  |  |  |  |  |  |  |  |  |  |  |  |
|  |  |  |  |  |  |  |  |  |  |  |  |
|  |  |  |  |  |  |  |  |  |  |  |  |
|  |  |  |  |  |  |  |  |  |  |  |  |
|  |  |  |  |  |  |  |  |  |  |  |  |

1. **Consultations (continued)**

| **Date of consultation** | **Type of Consultation** | | | | | **Health Professional seen** | | | | **Was this consultation about eczema? (tick one box)** | |
| --- | --- | --- | --- | --- | --- | --- | --- | --- | --- | --- | --- |
|  | **Did not attend booked appointment (DNA)** | **Face to face appointment** | **Telephone/ video / e-consultation** | **Home visit** | **Out of Hours / Walk-In Centre** | **GP** | **Practice Nurse** | **Nurse Practitioner** | **Health Visitor, Paramedic, Pharmacist, other** | **Yes** | **Not Sure** |
|  |  |  |  |  |  |  |  |  |  |  |  |
|  |  |  |  |  |  |  |  |  |  |  |  |
|  |  |  |  |  |  |  |  |  |  |  |  |
|  |  |  |  |  |  |  |  |  |  |  |  |
|  |  |  |  |  |  |  |  |  |  |  |  |
|  |  |  |  |  |  |  |  |  |  |  |  |
|  |  |  |  |  |  |  |  |  |  |  |  |

| **Date of consultation** | **Type of Consultation** | | | | | **Health Professional seen** | | | | **Was this consultation about eczema? (tick one box)** | |
| --- | --- | --- | --- | --- | --- | --- | --- | --- | --- | --- | --- |
|  | **Did not attend booked appointment (DNA)** | **Face to face appointment** | **Telephone/ video / e-consultation** | **Home visit** | **Out of Hours / Walk-In Centre** | **GP** | **Practice Nurse** | **Nurse Practitioner** | **Health Visitor, Paramedic, Pharmacist, other** | **Yes** | **Not Sure** |
|  |  |  |  |  |  |  |  |  |  |  |  |
|  |  |  |  |  |  |  |  |  |  |  |  |
|  |  |  |  |  |  |  |  |  |  |  |  |
|  |  |  |  |  |  |  |  |  |  |  |  |
|  |  |  |  |  |  |  |  |  |  |  |  |
|  |  |  |  |  |  |  |  |  |  |  |  |
|  |  |  |  |  |  |  |  |  |  |  |  |

1. **Please record all eczema related correspondence related to HOSPITAL or CLINIC or A&E attendance during the date period listed:**

| **Date** | **From**  ***(e.g. GP Surgery, Allergy Clinic)*** | **To**  ***(e.g. Dermatology, Paediatrician)*** | **Brief Summary**  ***(e.g. new referral, follow-up, discharge)*** |
| --- | --- | --- | --- |
|  |  |  |  |
|  |  |  |  |
|  |  |  |  |
|  |  |  |  |
|  |  |  |  |
|  |  |  |  |
|  |  |  |  |
|  |  |  |  |
|  |  |  |  |
|  |  |  |  |
|  |  |  |  |
|  |  |  |  |
|  |  |  | ` |

**Please scan and send this form to** [**eco@soton.ac.uk**](mailto:eco@soton.ac.uk) **(preferred option if possible please) or return by post using the following FREEPOST address.**

ECO STUDY

FREEPOST

[Address]

**Thank you!**

**Any queries, please email [email address] or phone [Number]**

If you need any additional space to record prescriptions or consultations, please provide details below:

Additional space to record prescriptions or consultations if required:
